# Pompe disease: pathogenesis, molecular genetics and diagnosis

**DOI:** 10.18632/aging.103794

**Published:** 2020-08-03

**Authors:** Simona Taverna, Giuseppe Cammarata, Paolo Colomba, Serafina Sciarrino, Carmela Zizzo, Daniele Francofonte, Marco Zora, Simone Scalia, Chiara Brando, Alessia Lo Curto, Emanuela Maria Marsana, Roberta Olivieri, Silvia Vitale, Giovanni Duro

**Affiliations:** 1 Institute for Biomedical Research and Innovation (IRIB-CNR), National Research Council of Italy, Palermo, Italy

**Keywords:** Pompe disease, acid alpha-1,4-glucosidase, lysosomal storage disorder, glycogen, GAA

## Abstract

Pompe disease (PD) is a rare autosomal recessive disorder caused by mutations in the GAA gene, localized on chromosome 17 and encoding for acid alpha-1,4-glucosidase (GAA). Currently, more than 560 mutations spread throughout *GAA* gene have been reported. GAA catalyzes the hydrolysis of α-1,4 and α-1,6-glucosidic bonds of glycogen and its deficiency leads to lysosomal storage of glycogen in several tissues, particularly in muscle. PD is a chronic and progressive pathology usually characterized by limb-girdle muscle weakness and respiratory failure. PD is classified as infantile and childhood/adult forms. PD patients exhibit a multisystemic manifestation that depends on age of onset.

Early diagnosis is essential to prevent or reduce the irreversible organ damage associated with PD progression. Here, we make an overview of PD focusing on pathogenesis, clinical phenotypes, molecular genetics, diagnosis, therapies, autophagy and the role of miRNAs as potential biomarkers for PD.

## INTRODUCTION

Lysosomal storage disorders (LSDs) are a subgroup of inherited diseases caused by inborn errors of metabolism [1, 2]. In LSDs, lysosomal enzymes are impaired and their functional deficit leads to substrate storage [3]. The catabolic role of lysosomes consists in breaking down and recycling of several substrates such as sphingolipids, glycogen, glycosaminoglycans, and proteins [4]. Different acidic hydrolases, such as glycosidases, lipases, sulfatases, phosphatases, peptidases and nucleases are involved in the lysosomal catabolic processes [5]. Pompe disease (OMIM # 232300) or glycogenosis type II or acid maltase deficiency is a rare, chronic and muscle-weakening, often fatal neuromuscular disease [6–8]. PD was described, for the first time, in 1932 by the Dutch physician Joanne Pompe in a 7-month-old child with general muscle weakness, who died from idiopathic cardiac hypertrophy. The association of the disease’s symptoms with the glycogen storage in all tissues was the first crucial observation [9]. In 1954 this disorder was classified as type II glycogen storage disease, but the correlation between this disorder, lysosomal storage, and enzymatic deficit was made in 1963 when the biochemist Hers discovered acid maltase [10]. This enzyme hydrolyses the glycogen into glucose at acid pH. In the same period, a deficit of acid maltase and a storage of glycogen in lysosomes were observed in PD patients; thus PD became the first disease classified as LSDs, which is a group of 50 disorders [11].

**Figure 1:**
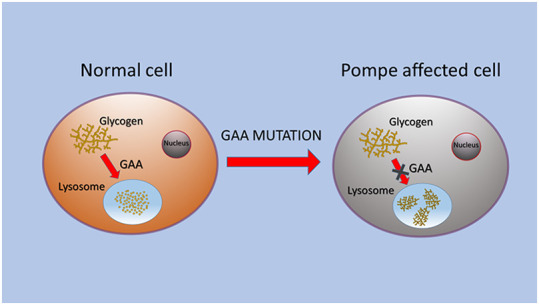
**Schematic representation of GAA alteration that caused glycogen storage in lysosomes of PD cells.**

PD is caused by a partial or total deficiency of acid alpha-glucosidase (GAA), which induces glycogen storage (Figure 1). Glycogen is an intracellular polymer of glucose residues linked by α 1→4 bonds in linear chains, and branches connected with α 1→6 bonds at branch points. GAA is synthesized as a membrane bound precursor, catalytically inactive, with an amino-terminal signal peptide. GAA precursor is sequestered in endoplasmic reticulum [9] where it is *N*-glycosylated, in seven glycosylation sites [12, 13]. The sugar chain is modified in Golgi complex and transported into lysosomes, where amino and carboxyl termini are cleaved in a stepwise process. The phosphorylation of the mannose residues induces enzyme transport to lysosomes via mannose 6-phosphate receptor, and in this organelle GAA hydrolyses the α 1→4 glucosidic bond in glycogen at acid pH [14, 15]. GAA contains five domains: N1 includes residues from 80 to 136, N2 from 137 to 346, C1 from 727 to 820, C2 from 821 to 952. The catalytic site is composed of residues from 347 to 726 with a barrel conformation. N2, C1 and C2 have β sandwich conformation [16, 17].

Glycogen is an important energy source during fasting, replaced in the fed state [18, 19]. A complex network of enzymes and regulatory proteins controls glycogen synthesis and degradation. The glycogen metabolism is also affected by mutations in genes encoding enzymes not involved in the classical metabolic pathways; this condition is referred to as “secondary glycogenosis” [20]. The deposits of glycogen induce a wide spectrum of clinical manifestations depending on storage site [17]. Recently, in LSDs, a growing number of studies reports a key role of epigenetic mechanisms such as DNA methylation, histone modifications, and microRNAs (miRNAs) [21–23]. In the era of precision medicine and liquid biopsy [24], the identification of new potential biomarkers in PD patients’ blood could be useful for an early diagnosis and monitoring of therapy.

In this review we make an overview of PD, focusing on pathogenesis, clinical phenotypes, molecular genetics, diagnosis, therapies, autophagy and the role of miRNAs as potential biomarkers for PD.

## Clinical phenotypes of PD

The clinical broad spectrum of PD depends on the age of onset. The severity of clinical manifestations, tissue impairment and age of onset correlate with the nature of mutations and the residual enzymatic activity levels [10]. PD is classified into two forms: Infantile Onset Pompe Disease (IOPD), considered as the classic form, and a late onset or non-classic form (Late Onset Pompe Disease, LOPD), which can occur at young or adult age [25–27]. IOPD is more severe than LOPD and begins at birth or within the first few months of life. It is characterized by cardiomyopathy and muscle weakness, and it can cause death in the first year of life [28]. A small percentage of patients show clinical signs with non-severe cardiomyopathy during the first year of life; this form of PD is referred to as non-classic IOPD [29, 30].

The signs and symptoms of IOPD are: delay or regression of motor development, alteration of intestinal tract with hepatomegaly and macroglossia, hypertrophic cardiomyopathy and ECG with short PR interval, high QRS complex voltage, arrhythmia and cardiorespiratory failure. PD children, suffered from “floppy baby” syndrome, are characterized by muscular hypotonia. PD patients, when affected by severe form, need a mechanical support to breathe.

LOPD differs widely depending on patient’s specific conditions, resulting in a progressive muscle weakness which is responsible for the motor difficulties and respiratory failure over time [31]. The signs and symptoms of LOPD involve: (I) skeletal muscles with skeletal myopathy, exercise intolerance, weakness of limb muscles and low back pain; (II) respiratory system with breathing failure, sleep apnea, dyspnea and respiratory infections [32]. The gastrointestinal symptoms, such as: macroglossia, hepatomegaly, are rare. LOPD patients can also show central nervous system injury with brain alterations. A cohort study demonstrated that in PD the prevalence of vasculopathy and dolichoectasia of vertebrobasilar system is higher than 50% and aneurysms are detectable in more than 10% of PD patients [33]. In LOPD patients, the most frequent symptoms at diagnosis are the musculoskeletal complications; 58,7% of patients manifest proximal muscles weakness of lower limbs [34]. PD incidence differs by ethnicity and geography; IOPD is characterized by a rapid progression, with a frequency of 1: 138,000 in white populations. PD incidence is estimated at 1 in 100,000 to 40,000 live births [35, 36] in the same population groups, but it is higher in specific population such as 1 in 15,000 in Taiwan [37] and 1 in 2000 in French Guiana [38], where a nationwide new-born screening (NBS) program was performed. Probably, in the countries where NBS is expected, the evaluation of PD incidence is more accurate than the others, in which only the diagnosed cases are reported, thus PD frequency might be underestimated.

## Molecular genetics of PD

PD is an autosomal recessive disorder, caused by a pathogenic variant in both copies of *GAA* gene. *GAA* is localized on long arm of chromosome 17 (17q25.2-q25.3), and consists of 20 exons: the first one is non-coding, the other 19 exons encode a protein of 952 amino-acids, with a molecular weight of 105-kDa [39, 40]. The first exon contains 5’ untranslated sequences and is separated from the second exon by a large intron. The first start codon, ATG, is located 32 nucleotides downstream from the beginning of exon 2 [41].

The mutational spectrum of *GAA* is very heterogeneous, genetic variants are often “private”, found only in a single family or in a small population [42, 43]. These variants can be: (I) point mutations, which can affect the protein functionality and stability or the splicing process, (II) small and large deletions and insertions. They cause the transcription of unstable mRNAs with consequences on: protein synthesis, post-translational modifications, lysosomal trafficking and in proteolytic nature of GAA. The most commonly reported missense mutations in PD occur in unexposed amino acid residues, causing structural misfolding, therefore PD can be considered a protein folding dysfunction [44].

In 2002, it has been reported that *GAA* variants were clustered in three critical regions of gene: exon 2, which contains the start codon; exon 10 and 11, which encode the catalytic site; and exon 14, which encodes for a highly conserved region of GAA protein [45]. However, several papers reported mutations in the whole gene [16, 46–48].

Pompe disease GAA variant database (http://www.pompevariantdatabase.nl/), last update in June 2019, reports 562 *GAA* variants, among these, 422 are disease-associated and 140 are considered Genetic Variants of Unknown Significance (GVUS). Moreover, the database provides information on variant severity [49].

**Figure 2:**
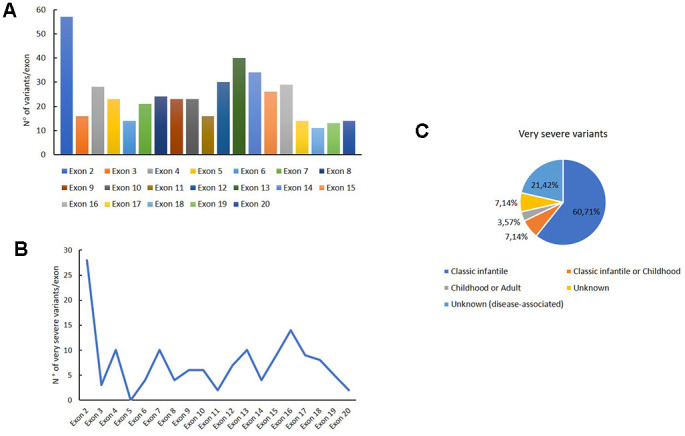
**Genetic variants distribution into *GAA* exons.** Distribution of variants for each exon (**A**); distribution of very severe variants for each exon (**B**); association of the very severe variants with PD phenotypes.

We carefully analysed the distribution of intronic and exonic mutations of *GAA* reported in this database. The variants distribution for each exon are reported in Figure 2A; as shown in the histogram, the major number of exonic variants are described in exon 2.

Figure 2B shows the distribution of very severe variants for each exon. These mutations are mainly reported in exon 2, in which the 49 % of all variants were associated with the very severe phenotype. Moreover, these variants are principally associated with a classical infantile form of PD, as shown in Figure 2C.

**Figure 3:**
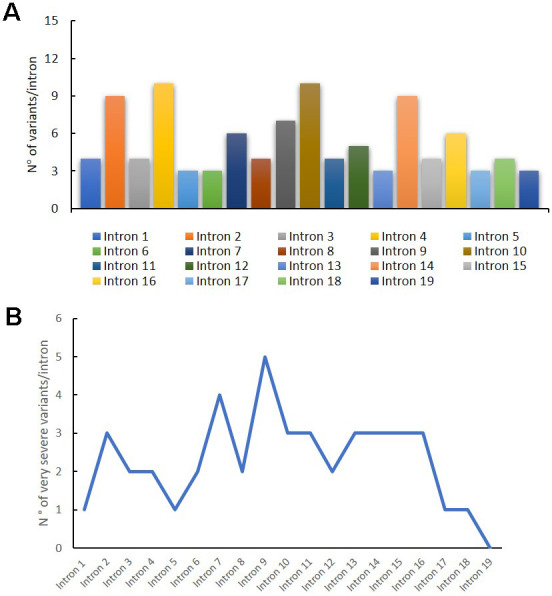
**Genetic variants distribution into *GAA* introns.** Distribution of variants for each intron (**A**); distribution of very severe variants for each intron (**B**).

In Figure 3A, we reported the variant distribution for each intron: as shown in the histogram, several variants are described in introns 2, 4, 10 and 14. Figure 3B shows the distribution of very severe variants for each intron. These mutations are mainly found in intron 9, in which all the reported variants are considered very severe (Figure 3B).

The most common mutation in Caucasian population is the intronic variant c.-32-13T>G (IVS1-13T>G). It causes a splicing defect that leads to exon 2 skipping, decreasing levels of synthesis (10-20%) of normal enzyme [40, 50]. Huie et al. described c.-32-13T>G mutation for the first time in a patient affected by LOPD [45, 51]. This mutation is located 13 nucleotides upstream of acceptor splice site of *GAA* in intron 1 and it is often associated with a second mutation in the other allele of *GAA*, which is usually more severe. The individuals homozygous for c.-32-13T>G were considered asymptomatic, but this hypothesis was proven to be incorrect. Patients with homozygous c.-32-13T>G showed myalgia, exercise-induced fatigue and increase of creatine kinase (CK) serum activity, a generic marker of muscle damage [52].

**Figure 4:**
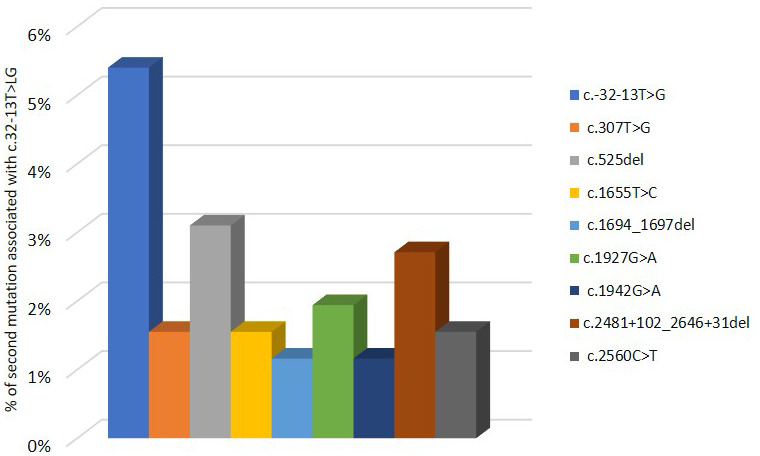
**Second mutation located in a second allele of *GAA* gene associated to c.-32-13T>G variant.**

Pompe GAA variant database indicates that c.-32-13T>G mutation was found in 258 patients and associated with different variants in the second allele of *GAA*, which is necessary to confirm PD diagnosis. As shown in Figure 4, the 5,4% of PD patients have c.-32-13T>G variant in homozygosis. The most described mutations in the other allele associated with c.-32-13T>G are located in exons 2, exon 14 and intron 17: in particular, 3,1% of the c.-32-13T>G is associated with a deletion in exon 2, c.525delT; 2,7% with a deletion in intron 17, c.2481+102_2646+31del; 1,95 % with a point mutation in exon 14, c.1927G>A (Figure 4).

*GAA* mutation’s distribution differs by ethnicity: in detail, del525T (exon 2) and c.925G>A (exon 5) are more frequent in Netherlands, but they were also found in other populations [53]. In Taiwanese patients the most common mutation is c.1935C>A (exon 14); while c.2560C>T (exon 18), is frequent in African American population.

The association of two variants: c.1726G>A (exon 12) and c.2065G>A (exon 15), often present *in cis*, are known to cause pseudo-deficiency of GAA. The c.1726G>A affects both amount of GAA and its catalytic activity, whereas c.2065G>A slightly reduces GAA functionality. Patients with these mutations in homozygosis have low levels of GAA activity without clinical signs of PD and they do not develop the disease [47, 54].

Recently, three new pathogenic mutations were reported in unrelated patients with LOPD carrying c.-32-13T>G mutation. Two of these variants were identified for the first time: the nonsense, c.2074C>T (p.Gln692X), and the missense mutation, c.1082C>G (p.Pro361Arg) found in exon 15 and 7 respectively. The deletion c.1910-1918del (p.Leu637_Val639del) located in exon 14, was previous considered as GVUS [55].

The frequency of mutations in homozygosis is low in Caucasian and Asian population, including Koreans and Chinese people [56, 57]. Since the symptoms of patients with LOPD are heterogeneous, the allelic diversity underlies the PD clinical heterogeneity and a different level of residual GAA enzymatic activity could deeply affect the disease phenotype [58].

In PD, as well as other genetic disorders, it is not easy to find a close correlation between genotype and phenotype. Up to 20% of mutations reported in *GAA* variant database are described without a strict correlation genotype/phenotype. PD patients with severe infantile form carry mutations that alter all forms of GAA causing low expression and enzymatic activity [47, 59]. The same mutations can be found in both infantile and late onset patients often with different incidence. Pittis et al. demonstrated that in two different groups of Italian patients, c.525delT variant was observed in 13,8% in IOPD and also in 3,8% in LOPD. The same authors reported different incidences of c.2237G >A in infants and adults, 3,4% and 10,3% respectively [59, 60]. A study on a large cohort of PD patients with a similar genotype reported that patients with c.-32-13T>G in combination with another mutation had different symptoms, suggesting the influence of secondary factors on disease progression. It was also shown that a deletion of gene encoding angiotensin-converting enzyme (ACE) caused an increase in type II muscle fibres and was associated to an early onset of PD, muscle pain, high levels of CK serum activity and a worse prognosis for patients with LOPD [60]. This study demonstrates that ACE polymorphisms are genetic factors able to modulate the clinical phenotype of PD patients.

## Diagnosis of PD

Physicians diagnose PD after the exclusion of the most common pathologies; thus, a dangerous and often fatal delay of PD diagnosis is noticed. In new-borns, early diagnosis is very important because, without treatment, death occurs within the first year of life. An analysis of Pompe data registry shows a diagnosis delay for all PD patients [8].

The median delay of diagnosis is 1,4 months in IOPD new-borns with cardiomyopathy and other symptoms developed during the first 12 months of life. In patients with the onset of symptomatology after 12 years old, the median delay is 6 years. In PD patients with the onset of symptoms during the first 12 months of life, without cardiomyopathy, the longest delay, 12,6 years, was reported. A similar delay was observed in PD patients with the symptom’s onset between 12 months and 12 years. Therefore, the disease should be diagnosed as early as possible [26, 36].

Recently, it was proposed a diagnostic algorithm, indicating that low GAA activity tested on Dried Blood Spot (DBS) should be confirmed by biochemical assays on different tissues and/or by a genetic analysis to complete the diagnosis [61]. PD European consensus, suggests that combination of enzymatic assay with gene sequencing is the gold standard for PD diagnosis [62].

### Enzymatic assay

The GAA activity analysis on DBS is a non-invasive, rapid, specific and reliable tool for PD diagnosis [48, 63].

GAA enzyme measurement is altered by the activity of maltase glucoamylase (MGA), another α-glucosidase active at acid pH that masks GAA deficiency. A strategy to selectively measure GAA, in presence of MGA, is a competitive inhibition using maltose or acarbose. Among these inhibitors, it was demonstrated that acarbose inhibited MGA better than maltose in DBS assay [64]. Recently, the use up to 2mM of 4-methylumbelliferyl-a-D-glucoside (4-MUG) in presence of acarbose in acidic conditions is indicated as a good method to test selective GAA activity by the report of the international consensus meeting on PD [62]. At acidic pH, the concentration of 3-9 μM of acarbose inhibits completely MGA without affecting GAA activity [64, 65]. Currently, two techniques are used to analyse DBS samples: fluorometric method and liquid chromatography-tandem mass spectrometry (LC-MS-MS). Both the two techniques are suitable to test GAA activity. A study on a large number of DBS demonstrated that GAA activity tested by MS is more accurate than fluorometric assay, to distinguish PD patients from individuals heterozygotes for one *GAA* mutation or with pseudo-deficit [66].

### Genetic analysis

*GAA* sequencing is used to confirm PD diagnosis and identify the pathogenic variants. *GAA* gene is highly polymorphic with several neutral variants. As aforementioned, the alterations of the gene include missense, nonsense and splice-site mutations, partial gene rearrangements, including small and large intragenic deletions and insertions. Sanger sequencing is the most common method to perform *GAA* gene analysis.

Since PD is an autosomal recessive disorder, PD patients have one mutation in homozygosis or 2 different mutations in compound heterozygosis. Multiplex ligation-dependent probe amplification (MLPA) analysis of *GAA* can be used to investigate the presence of large deletions [50], especially when a variant considered pathogenic or GVUS in heterozygosis were identified [67]. In patients with 2 different pathogenic variants, it is important to confirm the compound heterozygosis with a segregation study on relatives, in order to demonstrate that the two mutations are in two different alleles.

Recently, different NGS approaches for diagnosis of patients with skeletal muscle diseases were described [68, 69]. Savarese et al. analysed *GAA* and other genes associated with muscle diseases in a large cohort of undiagnosed patients with a wide spectrum of clinical phenotypes ranging from isolated hyper-CKemia to mild or severe muscular impairment, a variable age of onset and disease progression.

This mutational analysis identified pathogenic *GAA* variants in 10 patients and 7 relatives. Since the PD clinical signs overlap with the symptoms of other muscle disorders, *GAA* and other genes causing metabolic myopathies should be analysed in gene panels used for testing neuromuscular diseases, in order to identify PD patients that are potentially misdiagnosed [70].

In ‘t Groen and collegues indicated new molecular methods to validate PD diagnosis, when the standard procedures are insufficient. The authors performed extended molecular diagnostic analyses, such as a generic-splicing assay, minigene analysis, SNP array analysis, and targeted Sanger sequencing. These analyses allowed the identification of an exonic deletion, a promoter deletion, and a novel splicing variant located in 5’ UTR [71]. They reported, for the first time, pathogenic variants located in 2 critical regions for gene expression regulation: the promoter and 5’ UTR of *GAA* [49]. Nowadays, the aim of the researchers is to develop new tests for PD diagnosis able to detect new pathogenic variants and non-Mendelian genotypes that are not identified with the routine diagnostic assays.

### Unspecific analyses

Other unspecific laboratory parameters can be altered in PD patients, such as CK serum activity, aspartate (AST) and alanine (ALT) aminotransferase and lactate dehydrogenase; however, in PD patients these parameters can be unaffected. Therefore, the confirmatory tests (GAA activity and genetic analysis) have to be carried out in patients with a symptoms referable to PD [64]. A potential biomarker for glycogen storage diseases (GSD) is tetrasaccharide 6-α-D-glucopyranosyl-maltotriose (Glc4), because urinary excretion of Glc4 is increased in different clinical conditions associated with enhanced turnover or glycogen storage. Recently, a rapid ultraperformance LC-MS-MS assay was developed to characterize glycogen-derived tetrasaccharide in GSD [72]. Although this test is sensitive and precise for a presumptive diagnosis, it is not able to differentiate the GSD types. This assay should be used in combination with the standard enzymatic and genetic analyses to confirm of PD diagnosis. Few papers indicate that PAS-positive lymphocyte vacuoles can be used as diagnostic screening test for PD. The presence of glycogen-filled lysosomes in peripheral lymphocytes, detected by electron microscopy, and their vacuoles, observed by light microscopic in blood films of PD patients, was reported since 1977. Vacuolated lymphocytes were identified in blood films of patients with different pathologies, but the presence of periodic acid–Schiff (PAS)-positive vacuoles in lymphocytes was exclusively reported in PD patients, suggesting that their presence can be specific for PD [73, 74]. In PD patients, glycogen storage is found in lysosomes of all cells, including lymphocytes in peripheral blood. The detection of glycogen-filled vacuoles in lymphocytes by light microscopy on blood smears has been proposed as screening methods to identify PD patients among the individuals at risk of myopathy [73].

Muscle biopsy (MB) is used as an early diagnostic tool to evaluate muscle disease. The diagnostic value of MB in LOPD patients is rather limited, because different muscle groups and even fibers within the same muscle group, exhibit high variability. The visualization of a PAS positive vacuolar myopathy to identify LOPD can lead to false-negative results [75, 76]. However, histological identification of acid phosphatase-positive lipofuscin inclusions was suggested as a diagnostic marker for LOPD skeletal muscle. Lipofuscin accumulation caused by inefficient lysosomal degradation may in turn exacerbate both lysosomal and autophagic abnormalities. From the perspective of a clinician, MB is not reliable for diagnostic purposes, cannot be considered as a prognostic tool, and it exposes the patients to further discomfort and anesthesia risk. Considering the limits of MB, this procedure is not commonly used [77].

Since the limb-girdle weakness is a typical sign of the myopathy, the PD diagnosis can be challenging, especially without respiratory alterations. The patients with suspicion of PD often undergo electromyography (EMG) [78]. Early electromyographic studies indicated that electrical myotonia (EM) in axial muscles should raise the suspicion of PD, although it is also seen in other myopathies. Clinical and diagnostic findings in a cohort of 38 patients with LOPD showed that 71% of PD patients had a myopathic EMG pattern, half of these patients had spontaneous activity including complex repetitive discharges [79]. Another study on 37 patients with LOPD reported that twenty-eight (76%) had EM in at least one muscle, and in these patients the paraspinal and proximal limb muscles were the most commonly involved. The tensor fasciae latae (TFL) was equally sensitive to the paraspinals for EM. Some patients had EM identified in the diaphragm. Overall, these data indicated that three-quarters of LOPD patients display EM on EMG. The EM detected in the diaphragm of LOPD patients could be also due to the paraspinal muscles and TFL [80]. Although EMG is not a specific test for PD diagnosis, it helps to make a complete diagnosis.

The muscle magnetic resonance imaging (MRI) has an important role for the patients’ follow-up. Lollert et at indicated that the quantification of intramuscular fat in patients with LOPD by conventional MRI is useful for long-term follow-up of enzyme replacement therapy (ERT) [81].

For the follow-up of asymptomatic LOPD patients, it is important to detect muscle function alterations; although normal muscle function tests do not reveal the muscle structure integrity of these patients; muscle fiber loss and fatty replacement could have started without influencing the results of the tests yet. For this reason, quantitative muscle MRI (qMRI) has emerged as a valuable biomarker to follow up the progression of neuromuscular disorders. The qMRI is a non-invasive tool that quantifies the amount of fat in a muscle’s region of interest [82, 83]. In a study, 32 LOPD patients (22 symptomatic and 10 asymptomatic) underwent muscle MRI and were evaluated at the time of MRI and again after one year. Muscle MRI showed a significant increase of 1.7% in fat content of the thigh muscles in symptomatic LOPD patients. In contrast, there were no remarkable differences between muscle function tests in the same period of time. No significant changes either in muscle MRI in asymptomatic patients were observed over the year. To date muscle MRI is a useful tool for detecting changes in muscle structure in symptomatic LOPD patients and could become part of the current follow-up protocol in the clinical management [84].

To our knowledge, there are no papers that report the glycogen storage determination in blood by DBS to confirm PD diagnosis.

## Autophagy and PD

The deficiency of GAA activity is responsible for the intra-lysosomal storage of glycogen in all tissues especially in skeletal muscle and cardiac tissue; moreover, an increase of autophagic material is observed in skeletal muscle fibres [85]. PD was the first GSD linked to autophagy (self-eating). Autophagy is an evolutionary preserved catabolic process that leads to intracellular components degradation [86]. The autophagic process targets intracellular cytosolic components for lysosomal degradation and is important for sustaining cellular energy and metabolic homeostasis [87, 88].

Autophagy induces the formation of double-membrane vesicles, called autophagosomes, which incorporate cytoplasmic substances and then after fusion with lysosomes generate the autophagolysosomes, in which cargos are degraded by lysosomal enzymes [89].

The progressive storage of glycogen in lysosomes is responsible for a damage of their membranes, causing hydrolytic material dispersion in cytoplasm with the impairment of muscle contractile units. Autophagic pathway alteration caused further damage of muscle cells [90]. Recently, a specific form of autophagy of glycogen called glycophagy has been described. [91]. This process consists in degradation of cellular glycogen in autophagic vacuoles. Glycophagy plays a key role in maintaining glucose homeostasis and it is involved in glycogen sequestration, which is subsequently degraded by GAA. The breakdown of glycogen mediated by lysosomes triggers α-glucose release that can be rapidly used by cells [90]. The increase of autophagosomes and autophagy substrates, vacuolization and inappropriate lysosomal acidification were described in myotubes of patients and primary myoblasts of deficient mice, causing autophagy block. Moreover, autophagy influences GAA maturation and glycogen clearance [91–93].

Recently, it was reported that glycophagy modification is involved in PD and diabetic cardiomyopathy [94, 95]. Glycophagy can play an important role in pathological process of IOPD. In 2012 it was shown that stress-induced autophagy of endoplasmic reticulum, in IOPD patients, is induced by inactivation of AKT in fibroblasts. Two years later, Shemesh and colleagues observed a significant decrease in mTORC1 activation in *GAA*-knockdown myoblasts (C2C12) and *GAA*-deficient fibroblasts isolated from skin of IOPD patients. These data indicate that the decrease of mTORC1 activation could induce glycophagy.

Therefore, in IOPD this process could have a protective role that prevents the increase of glycogen-rich lysosomes. In contrast, in LOPD, the autophagy deregulation plays an important role in pathophysiological process. Raben et al., in adult patients, proposed that the massive storage of autophagic debris in muscles contributes to disease onset. Autophagy impairment was reported to affect vesicle trafficking and inhibit GAA maturation in LOPD; thus, glycophagy is involved in pathological process of LOPD. Literature data suggest that this mechanism could be a protective mechanism reducing glycogen-rich lysosomes storage in IOPD. The glycophagy modulation could be a new therapeutic strategy for IOPD.

Moreover, calcium homeostasis, oxidative stress and mitochondrial abnormalities can contribute to tissue damage that occurs in PD. Genotype-phenotype correlation studies on patients with the same *GAA* mutations showed several clinical manifestations caused by the interaction with other genetic and non-genetic factors. Some symptoms of PD patients overlap mitochondrial disorders [96]. The autophagy dysfunction is associated with inefficient mitophagy and reduced mitochondrial function [90] that can affect neuromuscular system. Mitochondria are essential for aerobic respiration by producing adenosine triphosphate (ATP), their function is controlled by mtDNA and nuclear genome but mtDNA alterations can be influenced by nuclear genome mutations or vice versa [52]. It was hypothesized that mtDNA interacts with *GAA*, but experimental data suggest that mtDNA variants might have a secondary role in PD pathogenesis. Understanding the role of mitochondria in PD pathogenesis can be potentially useful in development of new therapeutic strategies [97].

## miRNAs in PD

Epigenetic studies may be relevant to understand the wide clinical heterogeneity observed in monogenic disorders, as LSDs.

MiRNAs biogenesis pathway consists of different biochemical steps that convert the primary miRNA transcript (pri-miRNA) to mature miRNA biologically active. The mature miRNAs repress gene expression at specific target sites, which is dependent on complementarity between miRNAs and target sites. Each miRNA recognizes the 3’UTR of multiple mRNA transcripts and many miRNAs can recognize the same mRNA sequence [21, 98]. Ozsait and colleagues published the first correlation between LSDs and miRNAs [99]. Recently, the role of miRNAs in Fabry Disease (FD) was reported [100, 101]. Our research group identified a miRNA profile in plasma of FD patients, using high-throughput methodology. We selected miRNAs able to identify FD patients when compared to healthy controls. In particular, miR199a-5p and miR-126-3p are able to discriminate FD patients from control individuals with left ventricular hypertrophy. miR-423-5p and miR-451a could be suitable to study and monitor the cardiac involvement in FD patients [102].

Furthermore, the potential role of miRNAs in pathogenesis and progression of PD and as new biomarkers was also considered.

Using a high-throughput technology as NGS, miRNAs expression was studied in muscle and heart of a PD murine model and plasma of PD patients, in order to identify tools able to evaluate the patient clinical conditions and the response to treatments. The study started with a global analysis of miRNA expression profiles in skeletal muscle and heart of PD mouse model. miRNAs were altered in different tissues and age, suggesting modifications related to disease progression. It was also performed a small RNA-seq analysis in plasma of 6 patients, selected from 52 with IOPD and LOPD stored in Italian and Dutch biobanks. In this group of patients, 55 miRNAs were differentially expressed, among these, 16 miRNAs were differentially expressed both in tissues from PD mice and in patient’ plasma. In particular, miR-133a was selected for quantitative analysis in plasma of 52 patients. MiR-133a levels were significantly higher in PD patients than in healthy controls and correlated with phenotype severity. In IOPD, miR-133a levels are higher compared with LOPD. miR133a was decreased in three infantile patients that showed a clinical improvement after the beginning of ERT [22]. Circulating miRNAs can be considered potential additional biomarkers of PD progression and response to therapy.

In 2019, Carrasco-Rozas and colleagues performed miRNAs profile in serum of patients with LOPD. They analysed the expression of 185 miRNAs in serum of PD patients and controls and found 14 miRNAs differentially expressed between these two groups. Among these miRNAs, three were indicated as dystromirs: miR-1-3p, miR-133a-3p, and miR-206 showed different expression levels in serum samples from LOPD patients compared to controls. miR-1-3p, miR-133a-3p, and miR-206, increased in serum from LOPD patients, are involved in muscle regeneration [23].

Recently, it was reported the importance of including PD in differential diagnosis for patients with proximal muscle weakness. Twenty institutions in Latin America enrolled 2103 individuals with muscular dystrophy in whom a panel of 10 genes were investigated by NGS. Of these patients, 55,8% had genetic variants. Targeted intronic variants represented 2,9% of all pathogenic variants and GVUS; the major part of these intronic mutations was found in *GAA*. In the total population, less than half of samples showed no genetic variants, almost a third had a GVUS (29,8%), and 16% received a confirmed molecular diagnosis (homozygous or compound heterozygous). In particular, 9 patients received a confirmed molecular diagnosis of PD. The genotypes found in the newly identified LOPD patients are in agreement with the global experience, since the majority of these patients were heterozygous for the common splicing pathogenic variant IVS1-13T>G [103]. These data indicate that NGS allows the sequencing of several genes simultaneously and the improving of the diagnosis of Mendelian diseases with different phenotypes, such as PD [69, 104].

## Therapies for PD

### Enzymatic replacement therapies

The discovery of lysosomal enzyme uptake pathway mediated by mannose-6-phophate (M6P) receptor can lead to the cross-correction, indicating the possibility to replace a lysosomal enzyme by its supplementation in the extracellular media. In 2006 the ERT with recombinant human acid alpha-glucosidase (rhGAA) was approved for clinical use in patients with PD in Europe and US [105, 106]. PD prognosis has changed dramatically with the marketing authorization of ERT based on recombinant GAA. RhGAA is administered intravenously every two weeks at a recommended dose of 20 mg/kg, but higher dose regimens (up to 40 mg/kg) are recommended in IOPD patients.

ERT improves the cardiac and respiratory functions and contributes to extend the lifespan of IOPD patients. However, it is frequently associated with the development of neutralizing humoral immune responses against rhGAA that decreases treatment efficacy and survival. Skeletal muscle function is also enhanced by ERT. The clinical trials on LOPD indicates an improvement of muscle function as measured by 6-minute walk test whereas long-term studies show that respiratory function is only stabilized [105]. Nowadays, in order to overcome these limits, a second generation of rhGAA with higher affinity for the M6P receptors (25) is under evaluation in a phase III clinical trial. Another rhGAA called ATB20, carrying M6P and bis-M6P glycan residues, was developed and a clinical trial is ongoing in association with pharmacological chaperones (NCT03865836). Furthermore, a chimeric form of rhGAA containing a humanized Fab fragment derived from a murine antibody entered phase I/II clinical testing (NCT02898753) [107].

The limitations of therapy have encouraged efforts to enhance the efficacy of the current therapy and to develop new approaches including gene therapy.

### Gene therapy

A possible alternative to ERT is the gene therapy; since PD is a monogenic disorder, it is an ideal target for gene replacement strategies [105].

*In vivo* gene therapy consists of the administration of a gene delivery vector, viral or non-viral, directly into the cells of patient. Gene therapy is currently being developed for treatment of genetic disorders [17]. To date, the studies using adeno-associated virus (AAV) and retroviruses demonstrated the feasibility of gene therapy for PD [108]. AAV vectors were administered into the bloodstream to target, indirectly, the muscle, liver, or multiple tissues. AAV vectors can be also injected directly into the muscle or the cerebral ventricles to target the central nervous system [109, 110].

Recently, the production of AAV vectors in large scale and the positive results reported in preclinical studies of AAV delivery in neuromuscular diseases encouraged studying the AAV vectors containing muscle-specific expression cassettes for GAA transgene. The results showed an efficient clearance of glycogen storage in muscle and the improvement of the muscle and the cardiac and respiratory functions. One limitation of the systemic route to target muscles is the use of high doses of vector [111]. Moreover, muscle specific expression of GAA can increase the risk to develop anti-GAA antibodies causing a possible immunotoxicity. Another strategy to develop gene therapy for PD consists in the stable expression of GAA in liver. It was demonstrated that adenoviral GAA transfer mediates the cross-correction in skeletal muscles. The major limitation of this approach for PD is that hepatic gene transfer does not persist at long term [112].

In the era of genome editing, a potential therapeutic strategy for PD is based on the CRISP/CAS technology. This system relies on delivery of Cas9 protein and a RNA guide sequence to target and edit mutations in the genome. The gene can be edited by either non-homologous end joining (NHEJ) or homology-directed repair (HDR). CRISPR system using NHEJ would not correct the site-specific mutations found in PD, in which restoring a functional full-length GAA protein would be preferred. The site-specific corrections via HDR or other methods, such as base editors, would be necessary. HDR-mediated CRISPR strategies are not very efficient in muscle cells because DNA repair proteins, required for HDR, are low expressed [113, 114].

## Conclusion and perspectives

LSDs, caused by deficiency of lysosomal acid hydrolases, often lead to irreversible damage in cells and tissues, such as injuries to skeletal muscle, in PD. The affected organs can be excessively impaired at the time of diagnosis, hence it is necessary to reduce to diagnostic delay and start the treatment as early as possible.

GAA enzymatic activity assay is used as a first-line approach for PD diagnosis, if the enzymatic activity is low or borderline, genetic analyses need to be performed. Since PD is an autosomal recessive disorder, the genetic analysis in affected patients shows one mutation in homozygosis or 2 different mutations in compound heterozygosis. It is well-known that mutations are spread throughout *GAA*; therefore sequencing is performed in the whole gene. If GAA enzymatic activity is low and the sequencing reveals one pathogenic or GVUS mutation in heterozygosis, the genetic investigation should be completed with MLPA analysis of *GAA* to rule out deletions or insertions of several nucleotides, or with others extended genetic analyses. In patients with low enzymatic activity and 2 different pathogenetic variants, it is important confirming the compound heterozygosis with a segregation study on relatives, in order to demonstrate that the two mutations are in two different alleles.

Another important test to complete the diagnostic panel could be the determination of glycogen in blood. The accumulation of this substrate should be significantly higher in PD patients compared to healthy controls and subjects with pseudo-deficiency. To our knowledge, the determination of the glycogen storage in blood by DBS is not still performed to confirm PD diagnosis. The future aim for PD diagnosis is the improvement of quantitative assay for glycogen determination in blood. In other LSDs, LC-MS-MS [115, 116] is an accurate and reliable method to evaluate accumulated substrates such as globotriaosylsphingosine (LysoGB3) in Fabry disease [117]. LC-MS-MS might be used for glycogen storage determination.

The LSD study has made significant progress worldwide over the past three decades. The diagnosis of LSDs in asymptomatic or pre-symptomatic stage is considered a valid public health goal. PD inclusion in new-born screening (NBS) is becoming increasingly diffused [118–121]. Awareness of PD should avoid the diagnostic delay. The addition of LSDs to worldwide NBS will lead to an early diagnosis and avoid the diagnostic delay typical for these pathologies.

As aforementioned, it was considered the potential role of miRNAs as disease biomarkers in PD. The major challenge of researchers, for PD diagnosis, is to identify new markers, measurable, objective and not influenced by variance between investigators.

Using a high-throughput technology, miRNAs expression can be a tool to evaluate the patient clinical conditions and the response to treatments.
